# Prehospital care providers’ understanding of responsibilities during a behavioural emergency

**DOI:** 10.4102/sajpsychiatry.v27i0.1545

**Published:** 2021-01-27

**Authors:** Charnelle Stander, Peter Hodkinson, Enrico Dippenaar

**Affiliations:** 1 Division of Emergency Medicine, Faculty of Health Sciences, University of Cape Town, Cape Town, South Africa

**Keywords:** prehospital, responsibility, behaviour, emergency, mental healthcare

## Abstract

**Background:**

Prehospital emergency care providers are frequently called to assist with the management of mental healthcare users (MHCUs). The *Mental Health Care Act* no. 17 of 2002 regulates mental healthcare in South Africa, but the act fails to consider the responsibilities of prehospital emergency care providers in the provision of mental healthcare. Rather South African Police Services were given authority over the well-being of a MHCU in the prehospital setting.

**Aim:**

To investigate prehospital emergency care providers’ understanding of their responsibilities towards MHCUs and the community during the management of behavioural emergencies.

**Setting:**

The research was carried out at prehospital emergency care providers from the three main levels of care, currently operational within the boundaries of Pretoria, South Africa.

**Methods:**

A grounded theory qualitative study design was chosen using semi-structured focus groups for each level of prehospital emergency care – basic life support (BLS), intermediate life support (ILS) and advanced life support (ALS). Data from each focus group were collected through audio recordings and transcribed and analysed using a framework approach.

**Results:**

A total of 19 prehospital emergency care providers participated; two focus group interviews were performed for each level of care. The BLS focus groups each consisted of two participants. The ILS focus groups consisted of three participants each, and the ALS focus groups consisted of six and three participants. Four key themes were identified: perceptions of behavioural emergencies, responsibilities, understanding of legislation and barriers experienced.

**Conclusion:**

Participants placed high value on their moral and medical responsibilities towards MHCUs, which they described as ensuring the safety of themselves, MHCUs and the community; preventing further harm; and transporting MHCUs to an appropriate healthcare facility. There was a desire for revision of legislation, better education, skill development and awareness of mental healthcare in the prehospital emergency care setting.

## Introduction

South African prehospital emergency care is regulated by the *Constitution of the Republic of South Africa* No. 108 of 1996 (‘the constitution’), the *National Health Act* No. 61 of 2003, the *Health Professions Act* No. 56 of 1974 and guidelines provided by the Health Professions Council of South Africa (HPCSA).^1,2,3,4,5,6,7^ Within the South African prehospital emergency care setting, there are three main levels of care: basic life support (BLS), intermediate life support (ILS) and advanced life support (ALS). The full scope of practice for each level of care is described in the HPCSA clinical guidelines.^5,6,7^

Prehospital emergency care is a primary healthcare service and according to the *Health Professions Act* refers to:

[*T*]he rescue, evaluation, treatment and care of an ill or injured person in an emergency care situation and the continuations of treatment and care during transportation of such person to or between health establishments.^3,4^ (p. 1)

According to the constitution, no one may be refused emergency treatment, but guidelines for these emergency interventions are lacking.^1,8,9^ Confusion exists between laypersons and emergency care providers about what constitutes an emergency, as numerous definitions for ‘emergency’ are found within South African regulation.^8,10^

The *Mental Health Care Act* No. 17 of 2002 (MHCA) regulates mental healthcare in SA and focuses on deinstitutionalisation – the integration of mental healthcare into the general healthcare setting – which ensures that mental healthcare starts at the primary healthcare level.^11,12,13^ Deinstitutionalisation has led to an increase in the presentation of mental healthcare users (MHCUs) in the primary healthcare setting.^14,15^ The MHCA provides guidance to different primary healthcare providers on the provision of mental healthcare, but fails to recognise prehospital emergency care providers’ role in the provision of primary mental healthcare.^8,9,11^ According to Chapter 5 (Section 40) of the MHCA, the well-being of an MHCU is the responsibility of the South African Police Services (SAPS) in the prehospital setting.^11,16,17^ This means that SAPS members must make clinical decisions regarding an MHCU’s mental status without medical training or understanding of mental illness.^17^ An MHCU can present with changes in emotion, thinking and behaviour, which may cause impairment in their daily functionality – a behavioural disturbance, or in the case of a behavioural emergency, as defined by Kleespies, ‘a situation in which the impairment in a person’s thinking, emotions and behaviour places the person or bystanders in imminent physical harm’.^18,19,20,21,22^ According to all the aforementioned legislation, no treatment may be provided involuntarily – without consent.^2,3,11^ The only exception is during a behavioural emergency, and it is described in the MHCA, Chapter 3 (Section 9).^11^ Healthcare providers, which include prehospital emergency care providers, may provide involuntary care, treatment and rehabilitation to an MHCU, but it is unclear how this involuntary care and treatment should be provided in the prehospital setting.^8,9,11^

Even in countries such as Australia and the United Kingdom, where legislative support is provided to prehospital emergency care providers, they still lack the skills and knowledge to appropriately manage an MHCU and remain uncertain of their roles during the management of an MHCU.^23,24,25,26^ Studies have found that some prehospital emergency care providers considered behavioural emergencies a waste of emergency service resources.^25,26^

Prehospital emergency care providers are frequently called to assist with the management of an MHCU.^9,14,23,25^ The management of such a person is complex, as their human rights should be respected and their medical needs met whilst protecting the community. South African legislation is vague and provides little guidance on how to manage an MHCU in the prehospital setting. This study investigated a cohort of South African prehospital emergency care providers’ understanding of their responsibilities towards an MHCU and the community during the management of a behavioural emergency.

## Research methods and design

### Study design

A grounded theory qualitative research approach was chosen, using semi-structured focus group interviews.^27^

An interview guide was developed, which encouraged participants to introduce their own concepts and ideas. The focus group interviews were conducted by the principal investigator, an ALS prehospital emergency care provider, with the assistance of a qualitative research assistant. Separate focus group interviews were conducted for each of the three main levels of prehospital emergency care in SA, to recognise specific levels of care attributes and to compare differences between practitioners with varying levels of education and clinical experience. Focus group sizes were kept small, with no more than eight participants each, to allow in-depth discussion and equal opportunity for contributions.^28^

### Setting

Pretoria is situated in the northern part of Gauteng Province in South Africa. Both private and government emergency medical services (EMS) function within the city. Interviews were conducted at a convenient location for both the principal investigator and participants. Snacks and beverages were made available.

### Study population and sampling strategy

A convenience sampling approach was used, recruiting participants by distributing a research invitation on social media and amongst EMS organisations. A secured list of all emergency care providers that showed interest in participating was compiled, and those registered with the HPCSA, operational within the municipal boundaries of Pretoria, South Africa, and with at least 2 years’ clinical experience were included and invited to partake in the focus group interviews.

### Data collection

A total of six focus group interviews were held, two per level of care, with a total of 19 participants. The BLS focus groups each consisted of two participants. The ILS focus groups consisted of three participants each, and the ALS focus groups consisted of six and three participants. This was done to ensure data saturation and to explore or clarify issues raised in the first round. Data were collected using audio recording and observational field notes. To ensure anonymity, identifiers were allocated to each participant. The identifier consisted of three parts: firstly, the level of care of the participant – BLS (B), ILS (I) or ALS (A); secondly, participation in the second round of focus groups (indicated by a lowercase letter ‘b’); and thirdly, a number specific to one participant. The numbers did not always follow sequence in focus groups with less than eight participants.

### Data analysis

Focus group interviews were transcribed, which allowed for initial familiarisation with the data.^28,29^ Analytical notes on possible meanings and the introduction of bias were also made during transcription.^29^ Framework analysis was used to identify codes, which were arranged under a predefined structural code.^29^ Each question from the interview guide acted as a structural code: *Feelings and thoughts, Behavioural emergency meaning, Responsibility, Education and training, Systems, Police involvement, Legislation and guidelines* and *Mental capacity*.^29^ From these structural codes several themes were identified. The research assistant corroborated the analysis by common interpretation to ensure a level of reliability.

### Ethical consideration

Ethical approval was obtained from the Human Research Ethics Committee of the University of Cape Town (HREC REF 201/2018). All participants completed an informed consent form prior to the commencement of the focus group interview, provided verbal consent for audio recordings throughout and were reassured that their responses and opinions would remain anonymous.

## Results

### Participant demographics

Over the period from November 2018 to January 2019, four BLS, six ILS and nine ALS prehospital emergency care providers participated in the focus group interviews. An overview of the participants’ demographics per focus group is presented in Table 1 (It was discovered retrospectively that one BLS participant had only 1 year experience, but because of the low response rate this participant was included in the results).

**TABLE 1:** Participant demographics per focus group.

Focus group	Participant identifier	Gender	Years of experience	Sector
**Basic life support (*n* = 4)**	B07	Male	5	Private
	B08	Female	1	Private
	Bb01	Male	3	Private
	Bb02	Female	3	Private
**Intermediate life support (*n* = 6)**	I01	Male	**6**	Private
	I02	Male	5	Private
	I03	Female	3	Private
	Ib01	Male	3	Private
	Ib02	Male	17	Private
	Ib04	Male	3	Private
**Advanced life support (*n* = 9)**	A01	Male	9	Private
	A02	Male	9	Private
	A03	Male	17	Private
	A04	Female	25	Private
	A05	Male	5	Private
	A06	Male	11	Private
	Ab01	Male	10	Private
	Ab02	Male	5	Private
	Ab03	Male	2	Private

### Key themes

The structural codes were placed under four key themes, as shown in Table 2. Uncategorised codes were re-examined and assigned as best fit to either an existing structural code or a new category.

**TABLE 2:** Key themes and their structural codes.

Key themes	Structural codes
Theme 1 – Perception of behavioural emergency	Feelings and thoughts
	Behavioural emergency meaning
	Stigma and prevalence
Theme 2 – Prehospital provider’s responsibilities	General responsibilities
	Responsibilities when a patient is causing self-harm
	Responsibilities when a patient is causing harm to others
Theme 3 – Knowledge and understanding of current legislation	Police involvement
	Legislation and guidelines
	Mental capacity
Theme 4 – Barriers experienced during the management of a behavioural emergency	Education and training
	Response systems
	Laypeople and family members

#### Theme 1: Perception of behavioural emergencies

Participant Ab01 was of the opinion that the prevalence of behavioural emergencies in the prehospital setting had increased:

‘The World Health Organisation has predicted that this is going to grow … they’ve said [*that*] the number of incidences is definitively going to increase.’ (Ab01)

All participants reported negative feelings such as fear, frustration, apprehension, anger, aggression and uncertainty when being dispatched to a behavioural emergency. Participants felt that behavioural emergencies were time-consuming and emotionally and physically taxing:

‘… Apprehension. Because you do not know … and for me it’s like, I know to us it will be, ah, emotionally and mentally taxing. …’ (I01)

Participant Ib01 described prehospital emergency care providers as unsympathetic towards an MHCU:

‘… We are not a sympathetic group … you aren’t going to sympathise in that situation. …’ (Ib01)

Some participants reported positive feelings such as curiosity and empathy towards an MHCU. Participant A06 was the only one who reported feeling confident in managing an MHCU:

‘To my side of the West, it’s quite common … I won’t be scared or worried.’ (A06)

Participants described an MHCU behaviour as being ‘a deviation from norm’. An MHCU was described as unpredictable, presenting with behaviour that often fluctuates between a state of calmness and aggression and usually refusing treatment or transportation. The following mental health disorders were associated with MHCUs: post-traumatic stress disorder, acute psychosis, bipolar, depression, paranoia and schizophrenia. Participants were often called to manage situations involving panic attacks, self-harm, harm to others, self-neglect, domestic disputes, hallucination, suicide, overdose or an MHCU who needs someone to talk to:

‘… We are talking like all of the patients are like … cutting his business off, just saying “I want to die.” Those are the obvious ones … The, the patient I usually have a problem with … locked himself in his ho, house for three weeks. Literally lying on the floor wasting away, with severe sepsis. But he is still technically GCS fifteen … He is still looking at his telephone, looking at the laws going, “but you cannot touch me” … and you know for a fact, you can’t leave this oke here. He will die. …’ (A02)

#### Theme 2: Prehospital provider’s responsibilities

Participants felt that their own safety and that of other crew members took precedence. Once scene safety has been established, their next responsibility is to act as a neutral body, assess the situation and determine whether EMS or other resources are required:

‘Has the situation been uhm, neutralised … Is it an existing uhm, mental illness or not? Was it, was it just a normal fight uhm, domestic dispute? You know, those are all questions that we need to ask, because I don’t think all people in those situations [*need*] to go to a facility … we need to look at the whole situation and ask a lot of questions before we make decisions on what we going to do forward.’ (Ab02)

Participants viewed their responsibilities towards an MHCU as being to act in the best interests of the patient, provide appropriate medical treatment, prevent further harm and promote health:

‘… Treat the patient the best that you can. Prevent further harm and uhm, if there is harm, manage it appropriately uhm, just acting in the best interest of the patient. …’ (Ab03)

This is best done through a calm approach, removing the MHCU from the situation, listening attentively and where appropriate convincing the MHCU to be transported:

‘… Listen to the family as well as your patient. …’ (Ib02)

‘… To keep them comfortable and calm. …’ (B07)

Participants often reported lying to the MHCU to convince them to agree to transport and treatment:

‘If the patient is calm and at least speaking to you … change the scenario … If you can change your role in why you are there and mislead them. Because I’ve learnt deception works well with [*this*] type of patients.’ (Ib02)

An ALS participant, A05, felt strongly against this method. The participant suggested that an algorithm should be developed for the management of an MHCU, just as algorithms exist for the management of other medical emergencies such as anaphylaxis. Participant A03, who practised in the public sector, received an algorithm during orientation, but stated that the algorithm was too vague and basic:

‘I don’t think I, I ever had to lie to a patient … these are grey area patients. But I still treat them black or white … I don’t like resulting to the lying thing because by that point the white part is gone and the black part is coming … I think just as any other emergency … we have algorithms. …’ (A05)

Some participants stated that treatment for mental illness was outside their scope of practice and that it was not their responsibility to act as enforcers or mental healthcare providers. Intermediate life support participants in particular placed a high value on their responsibility as being more to report their findings on scene to the receiving facility to ensure that the MHCU receives adequate mental healthcare:

‘It is your responsibility to report it at the facility where you take the patient, but I don’t think it is our responsibility to sort out the mental illness.’ (Ib04)

The BLS and ILS groups saw their responsibilities as minor, during the management of a behavioural emergency, especially when managing an aggressive MHCU:

‘As a BLS our involvement is quite minimal. You are just an extra set of hands on scene. We don’t typically make decisions … That is up to the ALS or if the patient is not violent it is your ILS.’ (Bb02)

‘… It is not for us as intermediate life support practitioners to sedate, handle. We can try and defuse the situation. But, with regards to sedation of the aggressive … we can’t do much without ALS.’ (I01)

According to the ALS participants, they are a supportive role to SAPS during the management of an MHCU. Advanced life support participants realised that the main expectation of their role in the management of an aggressive MHCU was sedation. However, they did feel that BLS and ILS often asked for their assistance prematurely:

‘Crews called me for backup … “Bring your drugs, you are going to sedate.” “I’ll come up and assess this” … there was absolutely no indication for me to sedate him there and then.’ (A04)

#### Theme 3: Knowledge and understanding of current legislation

The participants appeared to have a good understanding of patient consent to treatment, as well as the requirement of mental capacity to provide consent. It seems that the Glasgow Coma Scale (GCS) as well as the participants’ own judgement as to whether a MHCU was grounded in reality were used to assess an MHCU’s mental capacity. However, participants did feel that they were not adequately trained to determining mental capacity:

‘… We can’t force them to stop hurting themselves … because if that guy says “I don’t want to” we are not allowed to touch him.’ (Bb01)

‘GCS 15 out of 15. Knows where they are. Know when. What time it is. Can answer all my questions … conscious of what’s going on (I02).’

‘… Trying to assess the patient’s mental state. That becomes a thing that is above our training.’ (Ib04)

Some participants knew of the MHCA, having heard of it through training or word of mouth, but showed little familiarity with the practical application of the act. Most participants reported having learnt through experience that when an MHCU does not consent and has the potential to cause harm, SAPS must be involved for involuntary transport of the MHCU to a facility that is equipped to observe and provide involuntary treatment to the MHCU:

‘Well, a lot of it comes from the medical health law … If we take that patient without them willingly come, coming with us … Then that’s why a lot of the times they would tell us to refer to SAPS.’ (I01)

‘… Those facilities most of the times would be [*names a few public government facilities*] … they need to be kept for that involuntary observation … for 72 hours.’ (Ib01)

One ALS participant, familiar with the MHCA, suggested that the current legislation and guidelines must be reviewed to clarify prehospital emergency care providers’ responsibilities during the management of an MHCU:

‘This is what’s happening. What are our rights? What are we obliged to do? How do we intervene here? … It’s possibly time to review the *Mental Health Care Act* and mandate such things.’ (Ab01)

#### Theme 4: Barriers experienced during the management of behavioural emergencies

Barriers that prevent participants from performing their perceived responsibilities as described above were also identified and encompassed issues with the current prehospital system, SAPS and family members.

Family members often expect prehospital emergency care providers to enter a scene and enforce treatment upon the MHCU, which leaves the MHCU apprehensive towards them:

‘Families threatening them, “[*t*]he ambulance is coming to take you away.” They [*are*] immediately apprehensive to talk to you or even allow you to treat them.’ (Ib02)

Difficulties experienced with the prehospital system included the dispatching system, scope of practice and inadequate education.

The dispatching system often provides inaccurate information around the type of emergency participants are being dispatched to, which make it difficult to adequately prepare:

‘… Where you get dispatched to a patient that fell … Yes, he fell. But that’s due to reasoning that he wants to jump from a building or a roof or. …’ (I02)

Education was a major concern amongst participants, who reported training in mental healthcare as inadequate, insufficient and varying across levels of care. Participants’ training focused more on medical emergencies, whilst knowledge gained on the management of behavioural disturbances was largely self-taught or through experience. Many participants showed interest in receiving additional training in mental healthcare as well as on the legislation relating to mental healthcare:

‘I think experience has prepared us, to kind of understand which situation has got the potential to go certain places and how to deal with it … most newly qualified people … I don’t think there’s enough things preparing them to deal with all of these situations.’ (Ab02)

South African Police Services involvement overall was described as reluctant and uncaring, and participants found SAPS members to lack understanding of the MHCA and mental illness. Participants Ab01 and A06 were of the opinion that SAPS members feared litigation, which may explain their reluctance. Participants thought that SAPS involvement should be limited to the safety of prehospital emergency care providers and the community:

‘… Our police are not equipped. And they don’t care about the well-being of the patient.’ (Bb01)

‘And from what I’ve picked up it’s also a fear of litigation … And that is also why the p, police just don’t want to get involved. But that also leaves us hanging.’ (Ab01)

Another barrier experienced was the lack of prehospital, SAPS and mental healthcare resources; participants provided suggestions to overcome these barriers, such as providing prehospital emergency care providers with the authority to transport an involuntary MHCU or having a mental healthcare expert with whom they can consult and make decisions regarding the MHCU’s treatment:

‘When we mandate certain things, this is the limitation of what paramedics can do and what police can do. This is what they are authorised to do, and then we mandate an expert being available for a telephonic consult. …’ (Ab01)

However, participant Ab03 did raise a concern about the appropriateness of prehospital emergency care providers managing an MHCU:

‘You require somebody that is specialised in that to deal with these patients, rather than someone like us. …’ (Ab03)

## Discussion

Prehospital emergency care providers in this study showed overwhelmingly negative feelings when dispatched to a behavioural disturbance, which is not unique to the South African setting.^9,14,15,23,30,31^ Participants experienced behavioural disturbances as emotionally and physically taxing but did not share the viewpoint, as described in the literature, that it was a waste of prehospital resources.^25,26^

Interestingly, the unsympathetic feelings of prehospital emergency care providers towards MHCUs has been experienced by MHCUs and is described by Rees et al.^30^ The results confirm that an MHCU can be perceived as a violent, overdosing or suicidal individual, who often refuses treatment and transportation.^8,25,30,31^ Safety was a major concern amongst the participants, who described the South African prehospital setting as unsafe; however, the participants were willing to act in the best interest of the MHCU and take responsibility for their medical and mental well-being. This finding is contradictory to local and international reports, which suggest that prehospital emergency care providers are unwilling to take steps to convince an MHCU to be transported or view an MHCU as an issue for social services.^8,25,26^

The study showed that prehospital emergency care providers are not only exposed to behavioural emergencies but also to MHCUs presenting with a wide range of behavioural disturbances.^23,25,26^

The responsibilities described by the participants during the management of an MHCU – treating injuries, preventing further harm and transporting the MHCU to an appropriate facility – are similar to the findings of other studies.^30,31,32^ The BLS participants saw their involvement as minimal in the management of MHCUs, whereas the ILS participants felt that when managing an aggressive MHCU, responsibility for the management of the MHCU falls upon ALS. It appears that at times BLS and ILS prehospital emergency care providers may assess a behavioural disturbance as a behavioural emergency and call for an ALS prehospital emergency care provider to sedate the MHCU, but once the ALS prehospital emergency care provider performs their own assessment no sedation is required. This finding substantiates Shaban’s findings that prehospital emergency care providers find it difficult to make accurate management decisions during these situations.^14,15,24,33^ One responsibility mentioned by participants, and not reported elsewhere, is reporting scene findings to the receiving facility to ensure that the MHCU receives appropriate care.

The HPCSA and MHCA provide no guidelines on the appropriate provision of involuntary care and treatment during the management of a behavioural emergency.^5,6,7,9,11^ The participants were unsure of what their responsibilities were and how to provide this involuntary care and treatment. They had a good understanding of consent to treatment but reported uncertainty in determining mental capacity. Healthcare providers must obtain informed consent from a compos mentis patient, before any treatment may be provided.^11,34^ However, available mental capacity assessment tools are designed predominantly for in-hospital use.^9^ According to the Medical Protection Society,^34^ mental capacity is defined as:

[*T*]he capacity to make decisions in light of information about the relevant risks, benefits and consequences of the proposed intervention, specifically being able to understand relevant information, appreciate the consequences of the situation and reason about the treatment. (p. 6)

Participants reported inadequate training in determining mental capacity, and it is supported that most participants used the GCS to determine mental capacity. This is an inappropriate use of the scale, which was intended to determine the level of consciousness following acute cerebral damage.^35^

Involving police services in the management of an MHCU has raised concerns such as the misuse of power and the violation of an MHCU’s basic human rights.^8,17,36,37^ As found in local and international studies, participants described police members reluctant to assist with the management of an MHCU who has the potential to cause harm.^8,25^ Participants felt that they were thus obligated to take responsibility for the well-being of MHCUs and often have to convince MHCUs to allow voluntary transport. This obligation prehospital emergency care providers feel that they have has also been described by Rees et al. and Prener et al.^25,30,31^ The participants believed that the current legislation should change to empower them to act in the best interest of MHCUs, and SAPS should only be responsible for scene safety. Another solution may be to have a mental healthcare expert available to both parties to assist in the decision-making process as to whether an MHCU requires involuntary assistance.

The study suggests that South African prehospital emergency care providers do not receive adequate training on the MHCA and learn largely through experience. Experience is what mostly guides decision-making processes in prehospital emergency care providers, but those who are inexperienced rely on protocols and guidelines.^14,24,33,38^ The lack in guidelines can lead to an inexperienced South African prehospital emergency care provider to make clinical decisions that may violate a MHCU’s human rights.^38,39^ This study identified a need for additional training and education in mental healthcare. The participants felt well prepared in the management of medical emergencies, but poorly prepared when managing a behavioural disturbance, which has also been reported worldwide.^15,23,25,26,30,31,32^

Family members often struggle to cope with MHCUs and phone for assistance.^36^ Negative feelings experienced by prehospital emergency care providers when dispatched to a behavioural disturbance; the description of a MHCU and behavioural emergencies and the difficulties experienced with police have been described in the international and local literature. The results unique to this study were the viewpoint that behavioural emergencies are not a waste of prehospital resources, but rather that there is no one else to take responsibility for the well-being of an MHCU in the prehospital setting.

## Strengths and limitations

This study is the first to explore how prehospital emergency care providers view their responsibilities during the management of a behavioural emergency and sets a baseline for further research on mental healthcare in the prehospital setting.

The study had a small and focussed sample size from a limited geographical area (Pretoria); thus the findings are not generalisable to the rest of SA. During the transcription of the first focus group discussion, it was noticed that on occasions participants’ comments were occasionally interrupted or completed by the principal investigator, especially when participants had difficulty expressing themselves. This might have led to the introduction of researcher bias by leading the answer.^40^ During the second focus group interviews, the principal investigator focused on limiting this type of researcher bias. Non-English-speaking participants may have had difficulty expressing their ideas, and it should have been considered to conduct focus group discussions in the participants’ home languages. The principal investigator had a working relationship with most of the participants. The relationship between BLS and ILS participants can be described as superior in the workplace but friendly and relaxed when not treating patients. Between ALS participants, the relationship is neutral as all have the same qualification. The principal investigator ensured a friendly environment and built rapport prior to the commencement of the focus group interviews to ensure that the participants felt comfortable in speaking and presenting their ideas.

## Recommendations

Further research on mental healthcare in the prehospital setting is required. Future studies should determine whether this study’s results can be generalised to SA. Future studies should investigate the possible legislative changes required to ensure that an MHCU is adequately managed in the prehospital setting and focus on the development of guidelines, adequate education techniques of these guidelines and how these guidelines will impact MHCUs and their interactions with prehospital emergency care providers.

## Conclusion

The prehospital emergency care providers in this study placed a high value on their moral and medical responsibilities towards MHCUs. They would have liked to have legislative support to fulfil their responsibilities towards MHCUs and desired better education, skill and understanding in mental healthcare. The BLS and ILS participants were of the opinion that most decisions made around the management of aggressive MHCUs were made by ALS practitioners. The South African legislation, prehospital clinical guidelines and training programmes need revision to clarify the responsibilities prehospital emergency care providers have during the management of MHCUs in the prehospital setting.
